# Hong Kong Chinese school children with elevated urine melamine levels: A prospective follow up study

**DOI:** 10.1186/1471-2458-11-354

**Published:** 2011-05-20

**Authors:** Alice PS Kong, Kai-Chow Choi, Chung Shun Ho, Michael HM Chan, Chun Kwok Wong, Eric KH Liu, Winnie CW Chu, Viola CY Chow, Joseph TF Lau, Juliana CN Chan

**Affiliations:** 1 Department of Medicine and Therapeutics, The Chinese University of Hong Kong, Prince of Wales Hospital, Shatin, Hong Kong SAR, China; 2 Li Ka Shing Institute of Health Sciences, The Chinese University of Hong Kong, Prince of Wales Hospital, Shatin, Hong Kong SAR, China; 3 The Nethersole School of Nursing, The Chinese University of Hong Kong, Shatin, Hong Kong SAR, China; 4 Department of Chemical Pathology, The Chinese University of Hong Kong, Prince of Wales Hospital, Shatin, Hong Kong SAR, China; 5 Department of Diagnostic Radiology and Imaging, The Chinese University of Hong Kong, Prince of Wales Hospital, Shatin, Hong Kong SAR, China; 6 Department of Microbiology, The Chinese University of Hong Kong, Prince of Wales Hospital, Shatin, Hong Kong SAR, China; 7 School of Public Health and Primary Care, The Chinese University of Hong Kong, Prince of Wales Hospital, Shatin, Hong Kong SAR, China; 8 Hong Kong Institute of Diabetes and Obesity, The Chinese University of Hong Kong, Prince of Wales Hospital, Shatin, Hong Kong SAR, China

## Abstract

**Background:**

In 2008, the outbreak of kidney stones in children fed by melamine-tainted milk products in Mainland China has caused major public concern of food safety. We identified Hong Kong school children with elevated urine melamine level from a community-based school survey in 2007-08 and reviewed their clinical status in 2009.

**Methods:**

In 2007-08, 2119 school children participated in a primary and secondary school survey in Hong Kong using a cluster sampling method. Urine aliquots from 502 subjects were assayed for melamine level. High urine melamine level was defined as urine melamine/creatinine ratio >7.1 μg/mmol. Subjects with high urine melamine level were invited for clinical evaluation in 2009 including urinalysis and ultrasound imaging of the urinary system.

**Results:**

The age range of this subcohort was 6 - 20 years with 67% girls (335 female and 167 male subjects). The spot urine melamine/creatinine ratio of the 502 urine aliquots ranged from undetectable to 1467 μg/mmol (median 0.8 μg/mmol). Of these, 213 subjects had undetectable level (42%). We invited 47 (9%) subjects with high urine melamine level for re-evaluation and one subject declined. The median duration of follow-up was 23.5 months (interquartile range: 19.8 - 30.6 months). None of the 46 subjects (28% boys, mean age 13.9 ± 2.9 years) had any abnormality detected on ultrasound study of the urinary system. All subjects had stable renal function with a median urine albumin-creatinine ratio of 0.70 mg/mmol (interquartile range: 0.00 - 2.55 mg/mmol).

**Conclusions:**

Hong Kong Chinese school children with high urine melamine levels appeared to have benign clinical course in the short term although a long term follow-up study is advisable in those with persistently high urine melamine level.

## Background

Since the outbreak of kidney stones in children fed with melamine-tainted milk products in Mainland China in early September 2008, the public concern of food safety has escalated to a new height [1]. Hong Kong is closely connected to Mainland China. Food supplies, immigrants and travellers from Mainland China are possible reasons for Hong Kong children to be affected by melamine-tainted food products. Moreover, melamine is widely used in plastics, dishware, laminates, glues and toy coating. Therefore, melamine is a potential food contaminant with public health hazards. Sporadic cases of melamine incident had been reported in Hong Kong and the incidence of renal involvement (echogenic renal foci) has been reported to range from 0.03% to 0.6% depending on selection criteria and methods of evaluation [2-4]. To date, there is no population data on melamine exposure and their possible health consequences.

In 2007-08, we conducted a territory wide survey to examine the prevalence of metabolic syndrome in Hong Kong youth. Using archived urine samples, we assayed the urine melamine levels in a subcohort and evaluated their clinical status after 2 years. We also explored the correlation between urinary melamine level and daily milk consumption in these school children.

## Methods

### Participants

Stored urine aliquots were collected from a territory-wide, population recruited cohort surveyed in 2007-08. The subjects and methodology have been described previously [5]. In brief, 2119 Hong Kong Chinese school children aged 6 - 20 years from 5 primary schools (804) and 6 secondary schools (1315) were randomly selected from all schools in Hong Kong using a cluster sampling method [5]. Informed written consents were sought from both the participants and their parents or guardians. The study was approved by the Joint Chinese University of Hong Kong-New Territories East Cluster Clinical Research Ethics Committee (CREC, Ref. No.: CRE-2006.136-T and CRE-2009.272). All participants were enquired about their habit of milk consumption using the question, "Are you drinking more than one cup of milk per day?" in a validated one-minute dietary questionnaire [6]. Spot morning urine specimens were collected from all agreeable participants during the field study for measurement of albumin-creatinine ratio (ACR). Albuminuria was defined as ACR > 3.5 mg/mmol [7]. A total of 502 urine aliquots were assayed for melamine level (funding was available for 502 melamine assays only). High urine melamine level was defined as urine melamine/creatinine ratio >7.1 μg/mmol [8]. Subjects with high urine melamine level were invited for clinical evaluation in 2009 including urinalysis and ultrasound imaging of the urinary system. Renal ultrasonography was performed by an experienced radiographer using the Philips ATL HDI5000 ultrasound machine. During ultrasound evaluations, both kidneys were carefully evaluated for the presence of renal stones, hydronephrosis or related renal scarring.

### Laboratory assays

Urine melamine was measured by a liquid chromatography tandem mass spectrometry (LC-MS/MS) method. Twenty μl of urine sample was added to 200 μl acetonitrile (ACN) containing 10 ppb stable isotope labeled melamine internal standard (^13^C_3_, ^15^N_3_-melamine, Cambridge Isotope Laboratories, Andover, MA, USA). The solution was vortex mixed before centrifugation at 16,000 g for 5 min and the clear supernatant was transferred to a sample vial, ready for LCTMS. The measurement was performed on a UPLC^® ^Waters Xevo TQ System (Waters, Milford, MA, USA). Calibrators, 10 - 1000 μg/l, were prepared by spiking appropriate amounts of melamine into a negative pooled urine sample. Five μl of calibrators/extracted melamine samples was injected into an ACQUITY UPLC^® ^BEH HILIC column (2.1 × 150 mm, 1.7 μm) which was kept at 45°C. Weak and strong wash solutions for UPLC^® ^were ACN and water, respectively. We used 50% ACN in water as the seal wash solution. Melamine and the internal standard were separated from matrix interference by a gradient program using mobile phase solutions of 10 mM ammonium acetate in water and 10 mM ammonium acetate in 97% ACN in water at a flow rate of 500 μl/min. For the mass analyzer, capillary voltage was optimized at 3.9 KV; cone voltage at 40 V and collision energy at 24 V. The source and desolvation temperatures were at 150°C and 500°C, respectively. Positive electrospray ionization tandem MS analyses were performed using *m/z *127-85 and 127-68 as quantitative and qualitative MRMs for melamine, respectively; and *m/z *133-89 as the MRM for the internal standard. The dwell time for each MRM was 50 msec. Both melamine and the internal standard eluted at around 3.5 min. Additional mobile phase gradient programming was used to remove matrix interference and recondition the column for the next analysis. Injection to injection time was 6 min. Quantitation was performed by the TargetLynx Manager of the Waters MassLynx 4.1 software. The limit of quantitation was 5 μg/l and the linearity was up to 10000 ug/l. Between-batch precision coefficients of variation for quality control samples (10, 100, 400 and 1000 ug/l) were < 10%. Recoveries for spiked standards into blank matrix at concentrations of 100, 400 and 1000 μg/l were >99%.

Urine albumin and creatinine were measured on a Roche Modular Analytics system (Roche Diagnostics GmbH, Mannheim, Germany). Urine albumin was measured by turbidimetry and urine creatinine was measured by kinetic Jaffe reaction using the standard reagent kits provided by the instrument manufacturer. Their analytical performances were within the manufacturer's specifications.

Urinalysis was performed by Multistix (Siemens urine test strips 10SG, Bayer). Microscopic examination was performed, by examining 60 μl of urine in microtitre plates using an inverted microscope, to look for red blood cells, casts and crystals in urine. Quantitative culture of urine was performed on Chromogenic medium (CPS ID3 [Biomerieus] plate using 10 μl standard loops incubated aerobically for 18 to 24 hr at 35°C.

### Statistical Analysis

Data were presented using appropriate descriptive statistics. Comparisons on baseline clinical and biochemical characteristics between those with and without high urinary melamine level (urine melamine/creatinine ratio >7.1 μg/mmol) were made using Pearson's Chi square test, Fisher's exact test, T-test and Mann-Whitney test, as appropriate. Association between daily milk consumption and melamine level was assessed using Mann-Whitney test and Pearson's Chi-square test, depending on the data format of melamine level. Spearman correlation coefficient was used to assess the correlation between urine ACR and urine melamine/creatinine ratio. All statistical analyses were performed using SPSS 17.0 (SPSS Inc., Chicago, IL). All statistical tests were two-sided and a P-value <0.05 was considered statistically significant.

## Results

Age of the study cohort ranged from 6 to 20 years with 67% girls (335 female and 167 male subjects). Table 1 shows the clinical and biochemical characteristics of the 502 school children recruited in this analysis. The spot urine melamine/creatinine ratio of the 502 school children ranged from undetectable to 1467 μg/mmol (median = 0.8) (Table 1). Of these, 213 school children had undetectable spot urine melamine/creatinine ratio (42%). Urine albumin ranged from undetectable to 207 mg/mmol. Median urine ACR was 0.70 mg/mmol (IQR: 0.45 - 2.01 mg/mmol). Age, body weight, body height and body mass index were associated with elevated urinary melamine level (Table 1). There was no significant correlation between urine melamine/creatinine ratio and ACR. There were 25 subjects with missing milk consumption data from the dietary questionnaire. There was no significant association between the milk consumption and urine analysis in 477 subjects with available data (Table 2).

**Table 1:** Baseline clinical and biochemical characteristics of the study sample (n = 502).

	All participants	Elevated urinary melamine level (urine melamine/creatinine ratio >7.1 ug/mmol)		
			
Characteristics	(n = 502)	No (n = 455)	Yes (n = 47)	p-value
Sex ^ψ^				
Male	167 (33.3%)	153 (33.6%)	14 (29.8%)	0.595 ^a^
Female	335 (66.7%)	302 (66.4%)	33 (70.2%)	
Age (years)	13.2 (3.0)	13.3 (3.0)	12.0 (2.8)	0.004 ^b^
Body weight (kg)	43.6 (11.8)	44.1 (11.7)	39.3 (12.1)	0.008 ^b,^*
Body height (cm)	152.3 (13.6)	152.8 (13.4)	147.4 (14.6)	0.010 ^b,^*
BMI (kg/m^2^)	18.5 (3.0)	18.6 (3.0)	17.5 (2.4)	0.026 ^b,^*
Weight status ^ψ^				
Normal	428 (85.3%)	384 (84.4%)	44 (93.6%)	0.193 ^c^
Overweight	51 (10.2%)	48 (10.5%)	3 (6.4%)	
Obesity	23 (4.6%)	23 (5.1%)	0	
Urine albumin-creatinine ratio (mg/mmol) ^†^	0.70 (0.45 - 2.01)	0.69 (0.44 - 3.23)	0.70 (0.50 - 1.39)	0.820 ^d^
Urine melamine/creatinine ratio (μg/mmol) ^†^, ^**§**^	0.76 (0.00 - 2.62)	0.52 (0.00 - 1.73)	13.21 (9.09 - 21.55)	<0.001 ^d^

Data marked with ^† ^were presented as medians (interquartile ranges) and with ^ψ ^as frequencies (%), all others were presented as means (standard deviations).

^a ^Chi square test; ^b ^T-test; ^c ^Fisher's exact test; ^d ^non-parametric Mann-Whitney test.

^**§ **^Undetectable urine melamine level was set to zero.

* There was no statistically significance between the two groups on body weight, body height, BMI and waist circumference after adjusting for age.

**Table 2:** Association between urine melamine level and daily milk consumption.

	Drinking more than a cup of milk per day #		
		
	No (n = 378)	Yes (n = 99)	p-value
Urine melamine (μg/l) ^**§**^	6.0 (0 - 25.0)	11.0 (0 - 33.0)	0.280 ^a^
Urine melamine/creatinine ratio (μg/mmol)	0.62 (0 - 2.33)	1.00 (0 - 3.19)	0.182 ^a^
Elevated urine melamine level (melamine/creatinine ratio >7.1 μg/mmol) ^ψ^			
No	347 (91.8%)	85 (85.9%)	0.072 ^b^
Yes	31 (8.2%)	14 (14.1%)	

Data marked ^ψ ^are presented as frequencies (percentages), all others are medians (interquartile ranges).

^# ^There were 25 subjects with missing milk consumption data.

^**§ **^Undetectable urine melamine level was set to zero.

^a ^Mann-Whitney test; ^b ^Pearson Chi square test.

Forty seven subjects (9%) with high urine melamine level were identified. One subject refused to come back for follow-up study. Median follow-up duration was 23.5 months (interquartile range, IQR: 19.8 - 30.6 months). Table 3 shows the clinical and biochemical characteristics of the 46 subjects (28% boys, mean age 13.9 ± 2.9 years) identified with elevated urine melamine level who returned for follow-up evaluation. None of them had any abnormality detected on ultrasound study of the urinary system. None of them recalled any significant urinary tract symptoms or had abnormalities on urinalysis.

**Table 3:** Clinical and biochemical characteristics of the 46 subjects with elevated urinary melamine levels, defined as urine melamine/creatinine ratio >7.1 ug/mmol.

	n (%)/mean (SD)/median (IQR))
Gender ^ψ^	
Male	13 (28.3%)
Female	33 (71.7%)
Age (years)	13.9 (2.9)
Body weight (kg)	47.9 (15.3)
Body height (cm)	155.9 (11.8)
BMI (kg/m^2^)	19.7 (4.5)
Urine total protein(g/l) ^ψ^	
<0.1*	35 (77.8%)
0.1 - <0.2	6 (13.3%)
0.2 - <0.3	2 (4.4%)
0.3 - <0.4	0
≥0.4	2 (4.4%)
Urine albumin-creatinine ratio (mg/mmol) ^†^	0.70 (0.00 - 2.55)
Abnormal ultrasound finding on the urinary system ^ψ^	0

Data marked with ^† ^were presented as medians (interquartile ranges) and with ^ψ ^as frequencies (%), all others were presented as means (standard deviations).

* minimum detectable level.

## Discussion

To our knowledge, our study is the first to explore the clinical course of subjects identified with elevated urine melamine level from a territory-wide survey. All participants were healthy volunteers without symptoms or known history of consumption of melamine-contaminated products. In agreement with a previous report of children aged 12 years or less known to have consumed milk products contaminated with melamine [2], we did not detect any major adverse renal outcomes in this cohort of older school children with elevated urine melamine level. Since albuminuria is known to be an early marker of renal damage, we investigated the association of urine albumin level with melamine level in subjects with high urine melamine level and did not find any significant association.

In this cohort, young age, low body weight and height, low body mass index were all associated with elevated urinary melamine level. Since young school children tend to have higher milk consumption, it is plausible that age may be the factor linking urinary melamine level and anthropometric parameters although we were not able to demonstrate a significant correlation between milk consumption and urinary melamine level, probably due to small sample size. In young children who were not breast-fed, milk products, particularly the powdered infant formula, are the major sources of nutrients in infants. In 2008, the discovery of heavy contamination of these milk products with melamine in Mainland China has raised alarm in the international community. Since 21 September 2008, China's Ministry of Health has reported that almost 40,000 children had been fed with melamine-tainted milk with 13,000 hospitalizations and acute renal failure in 104 children [1]. More than 52,000 children and infants had sought medical treatment after consumption of melamine-tainted formula in Mainland China with 4 reported deaths [9].

The melamine-tainted milk products were due to fraudulent adulteration for boosting the apparent protein content of milk [10]. Since melamine is not metabolized and rapidly eliminated in the urine, melamine and its structural analogues, such as cyanuric acid, may interact to form melamine-cyanurate crystals in cats and dogs [11]. In human and primates, which have much higher uric acid concentrations in the blood than cats and dogs, melamine-urate crystals are likely to form [11]. Animals fed with melamine developed kidney stones causing urinary tract obstruction [12]. To this end, deaths due to urinary tract stones and acute renal failure in infants and young children exposed to very high melamine level for prolonged period have been reported. Apart from being a food contaminant, melamine is also widely used in plastics, dishware, adhesives, and toys coating. Thus, it remains plausible that ingestion of melamine as environmental pollutants may be a silent health hazard to our youth population.

Our study has the strength that selection bias was unlikely due to the random nature of our school sampling and that the territory-wide survey was conducted before the outbreak of kidney stones related to melamine-tainted formula. Thus, potential recall bias in reporting milk consumption was not likely and might reflect the real picture of melamine ingestion from food and environmental pollution. In previous reports of renal incidents [2-4], subjects with known consumption of melamine-contaminated milk products or referred from designated outpatient clinics to special assessment centres were studied. These subjects might have symptoms or their parents might have increased alertness to seek medical attention. By contrast, our study has the advantage to identify subclinical or silent cases which might have been missed in previous studies.

There are several limitations of our study. First, only one random spot urine specimen was collected from the adolescents. School children with high melamine levels might have transient exposure to melamine-tainted food products. Whether prolonged or repeated exposure to consumption of melamine-contaminated food might lead to long term adverse renal outcome cannot be addressed by this short-term study of 2 years duration. Second, the sample size was relatively small with only 502 children being studied due to limited funding available to examine all urine aliquots for melamine level. Third, we did not record detailed food diary regarding the potential or known melamine-tainted milk products when urine was sampled in 2007-08.

## Conclusions

Hong Kong Chinese school children with high urine melamine levels appear to have benign clinical course in the short term. Periodic urine sampling for melamine level in a larger population will provide more complete information regarding the impact of prolonged exposure to melamine ingestion. In children and adolescents with persistently high urine melamine level, long term follow up is required to detect any adverse clinical effects.

## Competing interests

The authors declare that they have no competing interests.

## Authors' contributions

APSK prepared the proposal and supervised the study. APSK, KCC, CSH and JCNC interpreted the results, prepared the final manuscript and were responsible for data management and analysis. JTFL advised on data analysis. CSH, CKW, WCWC and JCNC contributed to the conception of the study and revision of the manuscript. CSH, MHMC, CKW and VC supervised the laboratory procedures. EKHL and WCWC provided technical support and supervised the imaging procedures. All authors read and approved the final manuscript.

## Pre-publication history

The pre-publication history for this paper can be accessed here:

http://www.biomedcentral.com/1471-2458/11/354/prepub
